# [(2*S*,3a*R*,6a*R*)-5-Oxohexa­hydro­furo[3,2-*b*]furan-2-yl]methyl acetate

**DOI:** 10.1107/S1600536813010313

**Published:** 2013-04-20

**Authors:** María González, Andrea Martínez, Marcos L. Rivadulla, Maria J. Matos

**Affiliations:** a Departamento Quimica Organica, Facultade de Quimica, Universidade de Vigo, E-36310 Vigo, Spain; b Departamento Quimica Organica, Facultade de Farmacia, Universidade de Santiago de Compostela, 15782 Santiago de Compostela, Spain

## Abstract

The title compound, C_9_H_12_O_5_, is a bicyclic lactone, presenting a 2,6-dioxabi­cyclo­[3.3.0]octan-3-one skeleton, which was obtained through an intra­molecular lactonization. The bicyclic lactone presents a *cis* ring-junction and a 1,5-*trans*-substituted tetra­hydro­furan. Both five-membered rings are in twisted envelope conformations with one of the fused C atoms as the flap. The dihedral angle between the mean planes of the bicyclic lactone residue, defined by the di­hydro­furan-2(3*H*)-one and the tetra­hydro­furan rings, is 69.5 (2)°. The atoms of the ester chain are coplanar [maximum deviation = 0.013 (2) Å]. The absolute structure was not determined.

## Related literature

For the stereoselective synthesis, applications and structures of related 2,6-dioxabi­cyclo­[3.3.0]octan-3-ones, see: Agrawal *et al.* (2006 ▶); Banda & Chakravarthy (2006 ▶); Paddon-Jones *et al.* (2001 ▶). For the biological activity of target compounds, see: Hayes *et al.* (2003 ▶). For the synthesis of chiral tetra­hydro­furans using l-malic acid, see: Álvarez *et al.* (2010 ▶). For pseudorotation parameters, see: Rao *et al.* (1981 ▶).
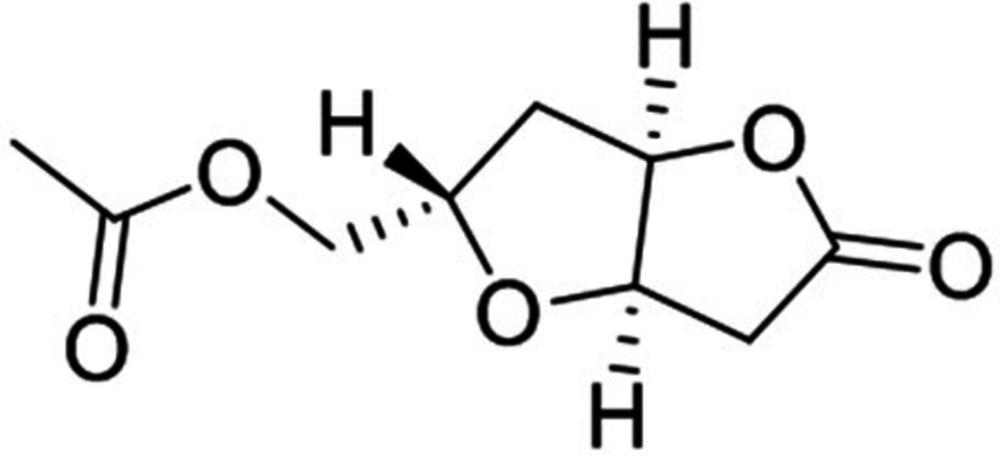


## Experimental

### 

#### Crystal data

C_9_H_12_O_5_
*M* *_r_* = 200.19
Monoclinic, 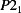

*a* = 10.015 (5) Å
*b* = 4.647 (3) Å
*c* = 10.904 (5) Å
β = 109.755 (7)°
*V* = 477.6 (4) Å^3^
*Z* = 2
Mo *K*α radiation
μ = 0.12 mm^−1^
*T* = 293 K
0.49 × 0.11 × 0.10 mm

#### Data collection

Bruker APEXII CCD diffractometer
Absorption correction: multi-scan (*SADABS*; Sheldrick, 2008 ▶) *T*_min_ = 0.602, *T*_max_ = 0.745
2551 measured reflections
1530 independent reflections
1313 reflections with *I* > 2σ(*I*)
*R*_int_ = 0.028

#### Refinement

*R*[*F* ^2^ > 2σ(*F* ^2^)] = 0.044
*wR*(*F* ^2^) = 0.097
*S* = 1.07
1530 reflections
128 parameters
1 restraint
H-atom parameters constrained
Δρ_max_ = 0.14 e Å^−3^
Δρ_min_ = −0.16 e Å^−3^

### 

Data collection: *APEX2* (Bruker, 2007 ▶); cell refinement: *SAINT* (Bruker, 2007 ▶); data reduction: *SAINT*; program(s) used to solve structure: *SHELXS97* (Sheldrick, 2008 ▶); program(s) used to refine structure: *SHELXL97* (Sheldrick, 2008 ▶); molecular graphics: *SHELXTL* (Sheldrick, 2008 ▶); software used to prepare material for publication: *PLATON* (Spek, 2009 ▶).

## Supplementary Material

Crystal structure: contains datablock(s) I, global. DOI: 10.1107/S1600536813010313/go2086sup1.cif

Structure factors: contains datablock(s) I. DOI: 10.1107/S1600536813010313/go2086Isup2.hkl

Supplementary material file. DOI: 10.1107/S1600536813010313/go2086Isup3.cml

Additional supplementary materials:  crystallographic information; 3D view; checkCIF report

## Figures and Tables

**Fig. 1.**
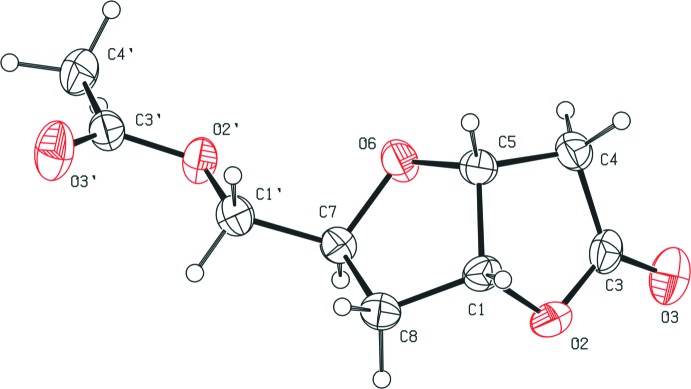
The molecular structure of the title compound with the atom-numbering scheme. Displacement ellipsoids are drawn at the 30% probability level.

## supplementary crystallographic information

### Comment

The 2,6-dioxabicyclo[3.3.0]octan-3-one skeleton is present in a number of natural products possessing diverse biological activities (Agrawal *et al.*, 2006; Banda *et al.*, 2006; Paddon-Jones *et al.*, 2001). We have described a new methodology for the synthesis of chiral tetrahydrofurans using *L*-malic acid (Álvarez *et al.*, 2010) as starting material. Using this strategy, a formal synthesis of (7*S*)-Hagen's gland lactones was achieved by an intramolecular lactonization protocol. These bicyclic lactones can be considered as potential bio-control agents for fruit-fly populations in different countries (Hayes *et al.*, 2003). Analyzing the crystallographic data, it is observed that both five-membered rings are in twisted envelope conformations Fig.1. The pseudorotation parameters P and τ(*M*), (Rao *et al.*, 1981), for the 5-membered ring containing O2 are P = 334.4 (4)°, τ(*M*) = 24.3 (2)°, for reference bond C5–C4, the closest pucker descriptor for this is an Envelope on C(5). The pseudorotation parameters P and τ(*M*) for the 5-membered ring containing O6 are P = 345.0 (4)°, τ(*M*) = 26.1 (2)°, for the reference bond C1–C8, the closest pucker descriptor for this is an Envelope on C1. The dihedral angle between the main planes of the bicyclic lactone residue is 69.52°. The dihedral angle between the tetrahydrofuran plane and the plane of the ester residue (defined as the C1'-O2'-C3'-C4' atoms) is 55.94°. The C1'-O2'-C3'-C4' atoms of the lateral chain are co-planar.

### Experimental

To a solution of (1*R*, 5*R*,7*S*)-7-hydroxymethyl-2,6-dioxabicyclo[3.3.0]octan-3-one (0.158 mmol) in pyridine (226 mL) was added Ac_2_O (149 mL) and the mixture was stirred at r.t. for 2 h. MeOH (3 mL) as added and the stirring continued for 20 min. The solvent was evaporated and EtOAc (5 mL) was added. The organic layer was washed with aq Cu_2_SO_4_ (3 *x* 5 mL), dried and the solvent was removed by rotary evaporation affording the title compound. It was then recrystallized using EtOAc. [mp: 87.1 – 89.9 °C; IR (NaCl, neat): 2917, 2849, 1771, 1740, 1462, 1370 cm^-1^; ^1^H-NMR (CDCl_3_): δ: 5.14 (t, 1H, J=3.7 Hz), 4.86 (s, 1H), 4.40 (d, 1H, J=5.5 Hz), 4.27 (d, 1H, J=11.9 Hz), 4.06 (dd, 1H, J=6.0 Hz, J=11.8 Hz), 2.76 (m, 2H), 2.44 (dd, 2H, J=5.3 Hz, J=13.9 Hz), 2.11 (s, 3H); ^13^C-NMR (CDCl_3_): δ: 175.53 (C=O), 170.85 (C=O), 84.34 (CH), 78.56 (CH), 76.41 (CH), 65.28 (CH_2_), 36.62 (CH_2_), 34.99 (CH_2_), 20.85 (CH_3_); HRMS: calcd for C_9_H_12_NaO_5_: 223.0577; found: 223.0575].

### Refinement

In all compounds H atoms were treated as riding atoms with C—H(primary), 0.97Å, C—H(secondary), 0.98Å with *U*_iso_ = 1.2Ueq(C)and C—H(methyl), 0.96Å. The positions of the methyl hydrogens was checked on a difference map. Since only C,H and O atoms were present in the molecule the quoted Flack parameter is meaningless and hence the absolute structure could not be determined.

### Crystal data

**Table:**

C_9_H_12_O_5_	*D*_x_ = 1.392 Mg m^−^^3^
*M**_r_* = 200.19	Melting point: 360.15(4) K
Monoclinic, *P*2_1_	Mo *K*α radiation, λ = 0.71073 Å
*a* = 10.015 (5) Å	Cell parameters from 1040 reflections
*b* = 4.647 (3) Å	θ = 2.4–24.1°
*c* = 10.904 (5) Å	µ = 0.12 mm^−^^1^
β = 109.755 (7)°	*T* = 293 K
*V* = 477.6 (4) Å^3^	Needle, colourless
*Z* = 2	0.49 × 0.11 × 0.10 mm
*F*(000) = 212	

### Data collection

**Table:**

Bruker APEXII CCD diffractometer	1530 independent reflections
Radiation source: fine-focus sealed tube	1313 reflections with *I* > 2σ(*I*)
Graphite monochromator	*R*_int_ = 0.028
φ and ω scans	θ_max_ = 25.1°, θ_min_ = 2.0°
Absorption correction: multi-scan (*SADABS*; Sheldrick, 2008)	*h* = −11→11
*T*_min_ = 0.602, *T*_max_ = 0.745	*k* = −5→5
2551 measured reflections	*l* = −13→12

### Refinement

**Table:**

Refinement on *F*^2^	Primary atom site location: structure-invariant direct methods
Least-squares matrix: full	Secondary atom site location: difference Fourier map
*R*[*F*^2^ > 2σ(*F*^2^)] = 0.044	Hydrogen site location: inferred from neighbouring sites
*wR*(*F*^2^) = 0.097	H-atom parameters constrained
*S* = 1.07	*w* = 1/[σ^2^(*F*_o_^2^) + (0.0377*P*)^2^ + 0.1292*P*] where *P* = (*F*_o_^2^ + 2*F*_c_^2^)/3
1530 reflections	(Δ/σ)_max_ < 0.001
128 parameters	Δρ_max_ = 0.14 e Å^−^^3^
1 restraint	Δρ_min_ = −0.16 e Å^−^^3^

### Special details

**Table:**

Geometry. All e.s.d.'s (except the e.s.d. in the dihedral angle between two l.s. planes) are estimated using the full covariance matrix. The cell e.s.d.'s are taken into account individually in the estimation of e.s.d.'s in distances, angles and torsion angles; correlations between e.s.d.'s in cell parameters are only used when they are defined by crystal symmetry. An approximate (isotropic) treatment of cell e.s.d.'s is used for estimating e.s.d.'s involving l.s. planes.
Refinement. Refinement of *F*^2^ against ALL reflections. The weighted *R*-factor *wR* and goodness of fit *S* are based on *F*^2^, conventional *R*-factors *R* are based on *F*, with *F* set to zero for negative *F*^2^. The threshold expression of *F*^2^ > σ(*F*^2^) is used only for calculating *R*-factors(gt) *etc*. and is not relevant to the choice of reflections for refinement. *R*-factors based on *F*^2^ are statistically about twice as large as those based on *F*, and *R*- factors based on ALL data will be even larger.

### Fractional atomic coordinates and isotropic or equivalent isotropic displacement parameters (Å^2^)

**Table:**

	*x*	*y*	*z*	*U*_iso_*/*U*_eq_	
C1	0.3092 (3)	0.6053 (7)	0.9619 (3)	0.0455 (7)	
H1	0.3648	0.4273	0.9825	0.055*	
O2	0.38327 (19)	0.8441 (5)	1.04241 (19)	0.0546 (6)	
C3	0.3280 (3)	0.9021 (7)	1.1363 (3)	0.0518 (8)	
O3	0.3786 (3)	1.0867 (6)	1.2155 (2)	0.0788 (7)	
C4	0.2033 (3)	0.7134 (8)	1.1221 (2)	0.0522 (8)	
H4A	0.1233	0.8252	1.1265	0.063*	
H4B	0.2263	0.5674	1.1896	0.063*	
O6	0.07106 (19)	0.7479 (6)	0.89421 (18)	0.0656 (7)	
C5	0.1713 (3)	0.5796 (7)	0.9901 (3)	0.0459 (7)	
H5	0.1407	0.3790	0.9887	0.055*	
C7	0.1203 (3)	0.8206 (7)	0.7894 (3)	0.0452 (7)	
H7	0.1309	1.0299	0.7865	0.054*	
C8	0.2656 (3)	0.6782 (8)	0.8200 (3)	0.0530 (8)	
H8A	0.2589	0.5060	0.7679	0.064*	
H8B	0.3329	0.8095	0.8034	0.064*	
C1'	0.0167 (3)	0.7196 (8)	0.6629 (3)	0.0487 (7)	
H1A	0.0550	0.7489	0.5932	0.058*	
H1B	−0.0025	0.5161	0.6677	0.058*	
O2'	−0.1124 (2)	0.8846 (5)	0.63829 (18)	0.0533 (5)	
C3'	−0.2165 (3)	0.8315 (7)	0.5261 (3)	0.0504 (7)	
O3'	−0.2036 (3)	0.6670 (6)	0.4477 (2)	0.0807 (8)	
C4'	−0.3452 (3)	1.0036 (8)	0.5127 (3)	0.0659 (10)	
H4E	−0.3893	0.9331	0.5725	0.099*	
H4D	−0.4104	0.9876	0.4252	0.099*	
H4C	−0.3192	1.2017	0.5318	0.099*	

### Atomic displacement parameters (Å^2^)

**Table:**

	*U*^11^	*U*^22^	*U*^33^	*U*^12^	*U*^13^	*U*^23^
C1	0.0348 (13)	0.0492 (18)	0.0516 (16)	0.0035 (14)	0.0136 (12)	0.0035 (14)
O2	0.0410 (10)	0.0622 (14)	0.0599 (12)	−0.0120 (11)	0.0162 (9)	−0.0041 (11)
C3	0.0467 (17)	0.057 (2)	0.0405 (16)	0.0046 (16)	0.0004 (13)	0.0065 (15)
O3	0.0824 (16)	0.0760 (18)	0.0616 (14)	−0.0014 (15)	0.0029 (12)	−0.0152 (14)
C4	0.0471 (16)	0.067 (2)	0.0430 (15)	0.0067 (15)	0.0164 (12)	0.0106 (14)
O6	0.0410 (11)	0.116 (2)	0.0426 (11)	0.0232 (12)	0.0172 (9)	0.0155 (12)
C5	0.0432 (15)	0.0489 (17)	0.0472 (15)	−0.0005 (14)	0.0173 (12)	0.0039 (14)
C7	0.0463 (15)	0.0496 (19)	0.0414 (14)	−0.0019 (14)	0.0173 (12)	−0.0020 (14)
C8	0.0455 (16)	0.067 (2)	0.0514 (17)	0.0022 (15)	0.0227 (13)	−0.0015 (15)
C1'	0.0545 (17)	0.0462 (16)	0.0460 (15)	0.0030 (15)	0.0179 (13)	−0.0009 (14)
O2'	0.0481 (11)	0.0631 (14)	0.0418 (11)	0.0103 (11)	0.0062 (9)	−0.0075 (10)
C3'	0.0558 (17)	0.0529 (19)	0.0402 (16)	−0.0051 (15)	0.0131 (14)	0.0017 (15)
O3'	0.0845 (17)	0.088 (2)	0.0559 (13)	0.0072 (14)	0.0055 (12)	−0.0251 (15)
C4'	0.0491 (18)	0.084 (3)	0.054 (2)	0.0063 (16)	0.0039 (15)	0.0061 (17)

### Geometric parameters (Å, º)

**Table:**

C1—O2	1.453 (3)	C7—C8	1.530 (4)
C1—C8	1.498 (4)	C7—H7	0.9800
C1—C5	1.518 (4)	C8—H8A	0.9700
C1—H1	0.9800	C8—H8B	0.9700
O2—C3	1.345 (4)	C1'—O2'	1.448 (3)
C3—O3	1.201 (4)	C1'—H1A	0.9700
C3—C4	1.490 (4)	C1'—H1B	0.9700
C4—C5	1.500 (4)	O2'—C3'	1.335 (3)
C4—H4A	0.9700	C3'—O3'	1.186 (4)
C4—H4B	0.9700	C3'—C4'	1.481 (4)
O6—C5	1.415 (3)	C4'—H4E	0.9600
O6—C7	1.430 (3)	C4'—H4D	0.9600
C5—H5	0.9800	C4'—H4C	0.9600
C7—C1'	1.494 (4)		
			
O2—C1—C8	111.3 (2)	O6—C7—H7	109.3
O2—C1—C5	104.6 (2)	C1'—C7—H7	109.3
C8—C1—C5	105.0 (2)	C8—C7—H7	109.3
O2—C1—H1	111.8	C1—C8—C7	104.3 (2)
C8—C1—H1	111.8	C1—C8—H8A	110.9
C5—C1—H1	111.8	C7—C8—H8A	110.9
C3—O2—C1	110.8 (2)	C1—C8—H8B	110.9
O3—C3—O2	120.5 (3)	C7—C8—H8B	110.9
O3—C3—C4	129.1 (3)	H8A—C8—H8B	108.9
O2—C3—C4	110.4 (3)	O2'—C1'—C7	107.6 (2)
C3—C4—C5	104.1 (2)	O2'—C1'—H1A	110.2
C3—C4—H4A	110.9	C7—C1'—H1A	110.2
C5—C4—H4A	110.9	O2'—C1'—H1B	110.2
C3—C4—H4B	110.9	C7—C1'—H1B	110.2
C5—C4—H4B	110.9	H1A—C1'—H1B	108.5
H4A—C4—H4B	109.0	C3'—O2'—C1'	116.4 (2)
C5—O6—C7	111.7 (2)	O3'—C3'—O2'	122.5 (3)
O6—C5—C4	110.4 (3)	O3'—C3'—C4'	125.4 (3)
O6—C5—C1	105.9 (2)	O2'—C3'—C4'	112.0 (3)
C4—C5—C1	104.3 (2)	C3'—C4'—H4E	109.5
O6—C5—H5	111.9	C3'—C4'—H4D	109.5
C4—C5—H5	111.9	H4E—C4'—H4D	109.5
C1—C5—H5	111.9	C3'—C4'—H4C	109.5
O6—C7—C1'	110.1 (2)	H4E—C4'—H4C	109.5
O6—C7—C8	106.5 (2)	H4D—C4'—H4C	109.5
C1'—C7—C8	112.2 (2)		
			
C8—C1—O2—C3	130.1 (2)	C8—C1—C5—C4	−141.0 (3)
C5—C1—O2—C3	17.2 (3)	C5—O6—C7—C1'	123.3 (3)
C1—O2—C3—O3	177.4 (3)	C5—O6—C7—C8	1.4 (4)
C1—O2—C3—C4	−3.3 (3)	O2—C1—C8—C7	−87.8 (3)
O3—C3—C4—C5	167.1 (3)	C5—C1—C8—C7	24.8 (3)
O2—C3—C4—C5	−12.1 (3)	O6—C7—C8—C1	−16.7 (3)
C7—O6—C5—C4	126.7 (3)	C1'—C7—C8—C1	−137.3 (3)
C7—O6—C5—C1	14.4 (3)	O6—C7—C1'—O2'	66.4 (3)
C3—C4—C5—O6	−91.7 (3)	C8—C7—C1'—O2'	−175.2 (2)
C3—C4—C5—C1	21.6 (3)	C7—C1'—O2'—C3'	177.6 (2)
O2—C1—C5—O6	92.8 (3)	C1'—O2'—C3'—O3'	−2.9 (4)
C8—C1—C5—O6	−24.5 (3)	C1'—O2'—C3'—C4'	178.0 (3)
O2—C1—C5—C4	−23.7 (3)		
